# Editorial: International Youth Day – pediatric rehabilitation from a global health perspective

**DOI:** 10.3389/fresc.2023.1234982

**Published:** 2023-08-16

**Authors:** Ana Paula Scoleze Ferrer

**Affiliations:** Faculty of Medicine, University of São Paulo, São Paulo, Brazil

**Keywords:** disability, rehabilitation, inclusion rehabilitation-, childhood disabilities, chronic disease, music-based interventions, health coaching, telerehabilitation

**Editorial on the Research Topic** International Youth Day - pediatric rehabilitation from a global health perspective

The World Health Organization (WHO) defines rehabilitation as “a set of interventions designed to optimize functioning and reduce disability in individuals with health conditions in interaction with their environment” (1). Globally, approximately 2.4 billion people are estimated to benefit from rehabilitation (2).

The demand for pediatric rehabilitation is growing, following an increase in the prevalence of chronic diseases in this age group, particularly in those patients classified as “children with special health needs” (3). In the United States, approximately 15%–20% of children with special health needs require rehabilitation (4, 5).

The benefits of rehabilitation are not only limited to the affected individuals but also extend to the society, as it promotes faster recovery, prevents complications, decreases chances of hospitalization, shortens the duration of hospitalization, favors the development of autonomy, and facilitates community participation, academic activities, return to work, and increased productivity (1, 2). In pediatric patients, these benefits are essential for their overall development and social participation and, in turn, improvement in their quality of life and that of their families.

According to the WHO, global rehabilitation needs have not been met because of multiple factors, including lack of public policies, low availability of resources, less investment, scarcity of technological resources, and a low number of qualified professionals (2). Thus, it is fundamental to conduct research and disseminate strategies aimed at making these services more accessible, qualified, and adaptable to the needs of the population.

The topic “International Youth Day—Pediatric Rehabilitation From a Global Health Perspective” brings forth a series of publications addressing this relevant theme from different perspectives. The topic ranges from local experiences of specific interventions for children with chronic conditions to tools aimed at ensuring the quality of remote care and telerehabilitation services. Furthermore, it introduces a platform that can support the management of resources at the health system level.

Sampaio reported experiences with a hospital humanization program using music for therapeutic rehabilitation and promotion of the well- being of children with chronic conditions and that of their families. The scope of music therapy has been proven; for example, it is an adjuvant therapy for patients with autism spectrum disorder (6). Despite growing evidence of the benefits of music therapy, there are limited publications on “how” music can be incorporated into pediatric rehabilitation. Thus, Sampaio's article is interesting because it describes experiences that can be developed by other professionals who have some training in music, since there is a shortage of music therapists in most countries, making this practice more accessible and applicable in other contexts.

Difficulty in accessing rehabilitation therapies is one of the main problems reported by the WHO, particularly in areas outside large urban centers. Offering remote services via telerehabilitation minimizes this disparity in the geographical distribution of available professionals and services (7). Particularly after the pandemic, remote access to health resources has become widespread, facilitating the performance of different rehabilitation modalities by people in need of isolation, with mobility issues due to health conditions, and/or socioeconomic reasons. Thus, online rehabilitation services ensure access to resources, favor continuity of care, allow remote monitoring, and encourage the active participation of patients and their families. However, to guarantee the quality and effectiveness of these services, it is essential that some assumptions are evaluated: safety, efficacy, quality of care, patient/family and provider satisfaction, costs, access and availability of services (7, 8).

Two articles on this topic have presented tools to ensure the quality of remote rehabilitation services. One article by Ogourtsova described TelereHUB-CHILD, an online tool that translates and updates existing knowledge in telerehabilitation, which facilitates evidence-based decision-making by health professionals, helps train parents, family members, and patients, and identifies gaps in existing knowledge. This report also described the steps involved in the development of this tool, which incorporates technological advances into care offered and includes the participation of the main stakeholders involved.

Owing to difficulties in access to and delays in receiving specialized care, health coaching programs have been disseminated to facilitate accessibility, but is very important to evaluate their effectiveness. Another study by Ogourtsova et al. described the CO-FIDEL program, a tool which evaluates the fidelity of Bright coaching, a Canadian program aimed at remotely supporting caregivers of children with suspected or delayed (9). Authors also assessed the viability and user satisfaction with the CO-FIDEL, providing data for developers of health coaching programs.

Hanson et al. conducted a descriptive and analytical study on the provision of mental health services for pediatric patients with disabilities. Since there is a growing demand for these services, and difficulties in access are observed in most countries, the study results can help healthcare managers identify the available services and streamline their provision.

Considering the importance of pediatric rehabilitation for the patients, their families, and society and the high rates of unmet needs (10), the topic “International Youth Day—Pediatric Rehabilitation From a Global Health Perspective” addresses various aspects related to the implementation of the WHO Rehabilitation 2030 initiative, translating practical experiences that can be applied in health care and supporting the formulation of public policies aimed at improving access to rehabilitation services.
